# Supplementary figures and images for: Comparative transcriptome analysis of albino northern snakehead (Channa argus) reveals its various collagen-related DEGs in caudal fin cells
Source: PLoS One. 2024 Dec 31;19(12):e0315996. doi: 10.1371/journal.pone.0315996 (PMC11687805; doi:10.1371/journal.pone.0315996)

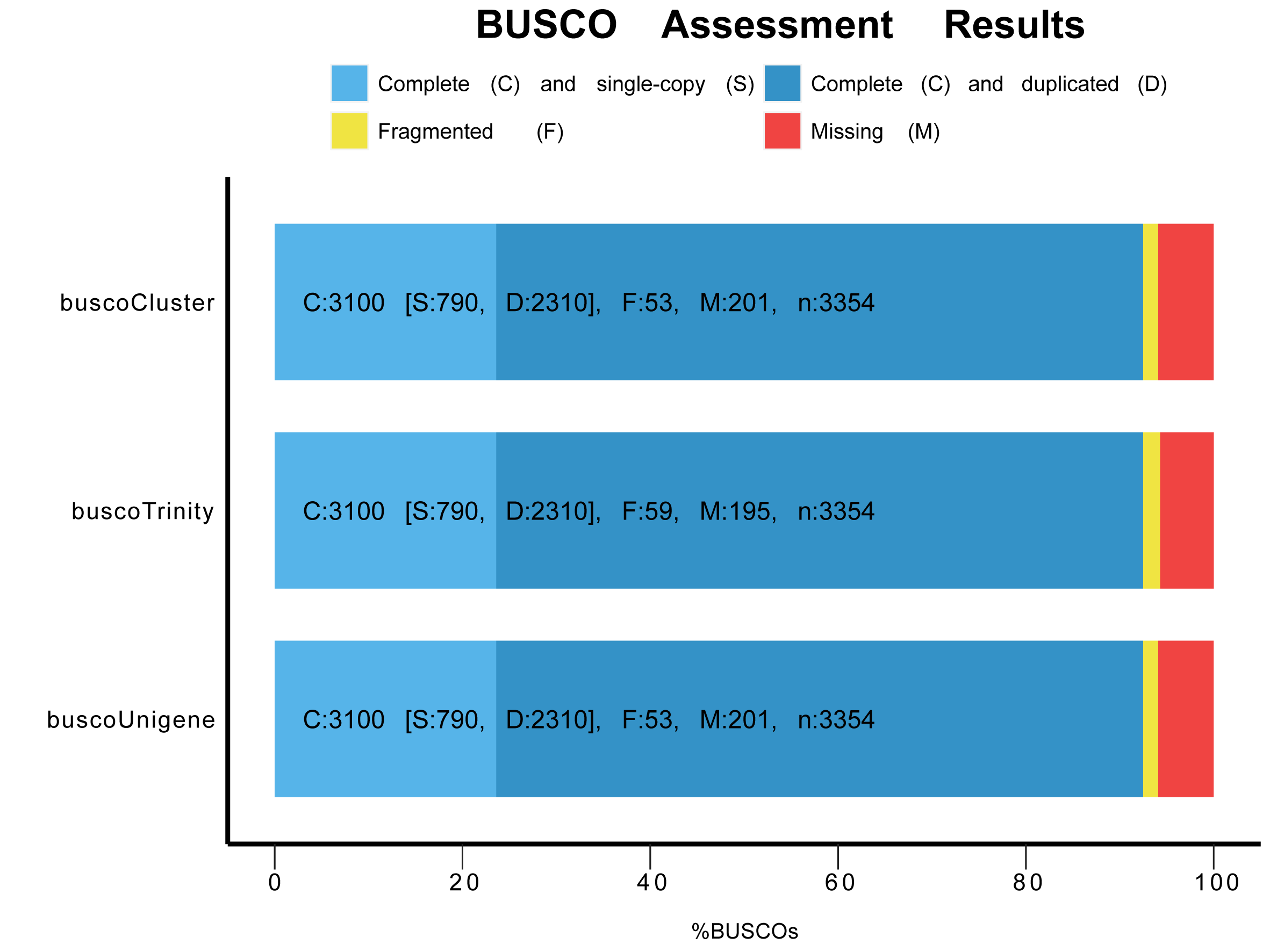

Supplement: S1 Fig — Light blue: complete (C) and single copy genes (S); dark blue: complete (C) and duplicated genes (D); yellow: fragmented genes (F); red: missing genes (M). (TIF) [file pone.0315996.s002.tif]

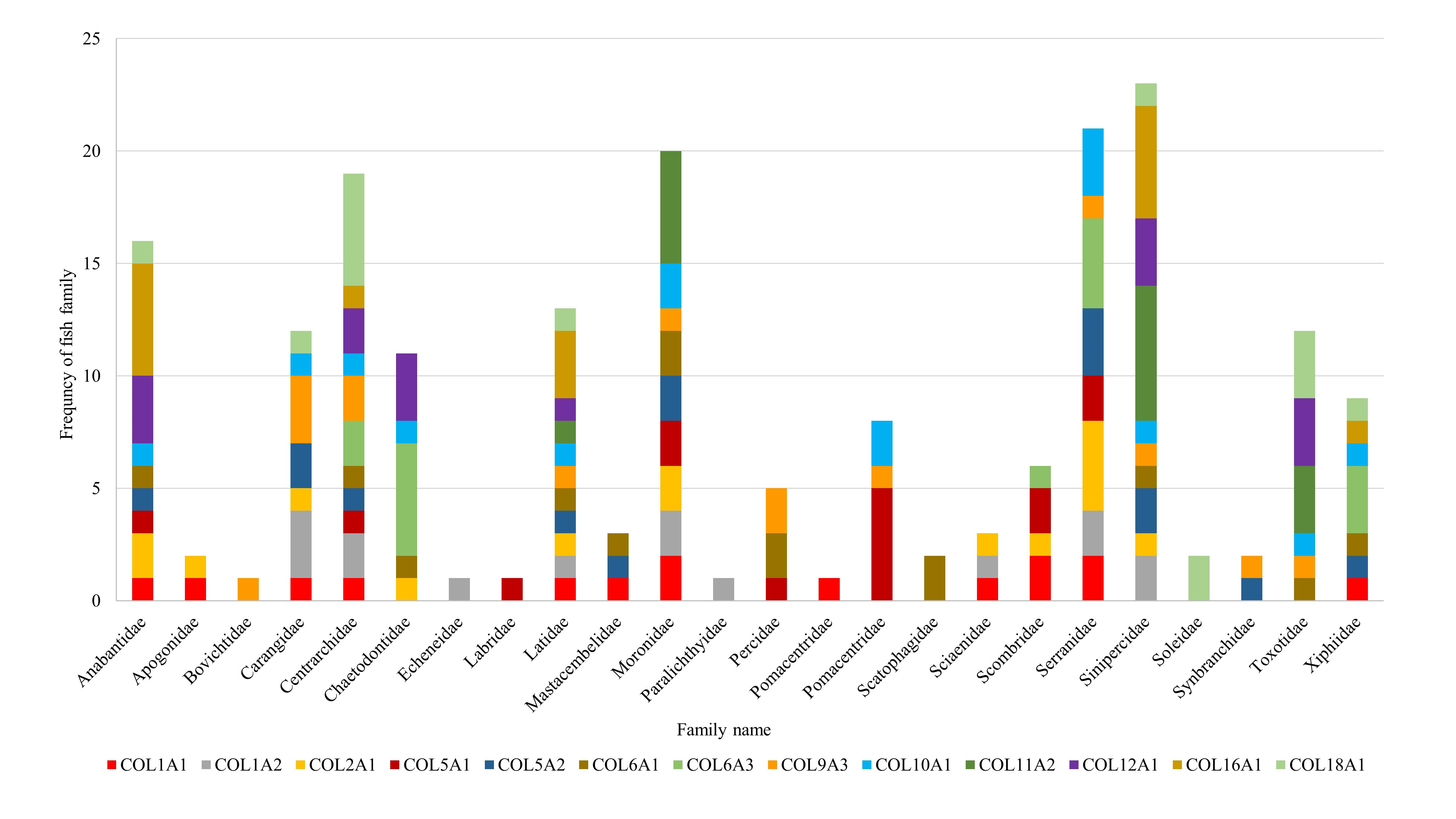

Supplement: S2 Fig — (TIFF) [file pone.0315996.s003.tiff]
