# Supplementary material for: Comparative transcriptome analysis of albino northern snakehead (Channa argus) reveals its various collagen-related DEGs in caudal fin cells
Source: PLoS One. 2024 Dec 31;19(12):e0315996. doi: 10.1371/journal.pone.0315996 (PMC11687805; doi:10.1371/journal.pone.0315996)
Supplement: Attachment — Submitted filename: Plose review.docx [file pone.0315996.s004.docx]

Dear Editor

Manuscript can be accepted in current form

Abstract

Abstract is explained in a clear and concise manner.

Keywords

I am of the opinion that if the keywords include words other than the words in the title, it may increase the citation and recognition of the article.

Introduction

The introduction was written in a way to cover the subject, the literature was used correctly and it was very descriptive.

Material Method

The material and method is sufficiently clear. Complete line 147 in manuscript file.

Result and Discussion

Under each heading, the subject is explained in such a way that no questions are left in mind and the literature is explained in a comprehensive manner.
