# Supplementary material for: Comparative transcriptome analysis of albino northern snakehead (Channa argus) reveals its various collagen-related DEGs in caudal fin cells
Source: PLoS One. 2024 Dec 31;19(12):e0315996. doi: 10.1371/journal.pone.0315996 (PMC11687805; doi:10.1371/journal.pone.0315996)
Supplement: Attachment — Submitted filename: Response to Reviewers.docx [file pone.0315996.s005.docx]

Response to Reviewers:

Thanks to the reviewers for the comments and suggested revisions to this manuscript. The authors responses are shared below in a table, and the manuscript is revised according to the track changes. The table has two sections: 1. For Academic editor and 2. For reviewers. Moreover, the lines mentioned in the table below are according to the revised manuscript.

| **Academic Editor** | **Comments** | **Response from Author** |
| --- | --- | --- |
| **1** | Please ensure that your manuscript meets PLOS ONE's style requirements, including those for file naming. The PLOS ONE style templates can be found at  https://journals.plos.org/plosone/s/file?id=wjVg/PLOSOne_formatting_sample_main_body.pdf and  https://journals.plos.org/plosone/s/file?id=ba62/PLOSOne_formatting_sample_title_authors_affiliations.pdf | The manuscript has been extensively revised as per PLOS One’s style requirement that inclues author page and reference style, figure and table legends and others. |
| **2** | Note from Emily Chenette, Editor in Chief of PLOS ONE, and Iain Hrynaszkiewicz, Director of Open Research Solutions at PLOS: Did you know that depositing data in a repository is associated with up to a 25% citation advantage (https://doi.org/10.1371/journal.pone.0230416)? If you’ve not already done so, consider depositing your raw data in a repository to ensure your work is read, appreciated and cited by the largest possible audience. You’ll also earn an Accessible Data icon on your published paper if you deposit your data in any participating repository (https://plos.org/open-science/open-data/#accessible-data). | Our data is deposited and the information is given in the ‘Data Availability Statement’. |
| **3** | We noticed you have some minor occurrence of overlapping text with the following previous publication(s), which needs to be addressed: https://linkinghub.elsevier.com/retrieve/pii/S2405844023021448 https://www.tandfonline.com/doi/abs/10.1080/10408398.2020.1751585  In your revision ensure you cite all your sources (including your own works), and quote or rephrase any duplicated text outside the methods section. Further consideration is dependent on these concerns being addressed. | Overlapping texts have been revised. We used online tools to check the plagiarism of the manuscript (introduction and discussion section) which revealed that the sectiosn were unique. |
| **4** | 4. In the online submission form, you indicated that the data supporting these findings will be available in online repositories. The names of the repository/repositories and accession numbers are as follows: Bioproject number PRJNA913664 and BioSample number SAMN32303246, which will be released on October 5, 2023. Before the above date, the raw data supporting the conclusions of this article will be made available by the authors upon reasonable request without undue reservation. All PLOS journals now require all data underlying the findings described in their manuscript to be freely available to other researchers, either 1. In a public repository, 2. Within the manuscript itself, or 3. Uploaded as supplementary information. This policy applies to all data except where public deposition would breach compliance with the protocol approved by your research ethics board. If your data cannot be made publicly available for ethical or legal reasons (e.g., public availability would compromise patient privacy), please explain your reasons on resubmission and your exemption request will be escalated for approval. | Our data is deposited and already published. The related information is given in the ‘Data Availability Statement’. |
| **5** | We notice that your supplementary figures are included in the manuscript file. Please remove them and upload them with the file type 'Supporting Information'. Please ensure that each Supporting Information file has a legend listed in the manuscript after the references list. | Supporting Information file has a legend listed in the manuscript after the references list. Files are uploaded in the ‘Supporting Information’ section in the system. |
| **6** | In my opinion and based on the reviewers` comments I suggest the deep revision of the paper. I would like to give the opportunity to the Authors to make a correction of their manuscript. The main concern is the methodology, while the second is that the conclusions are not supported by the results. Thus all these aspects should be improved. Please make a correction according to the attached comments of the Reviewers. | We have through revised every section in the manuscript as per review comments. |
|  | | |
| **Reviewer 1** | **Reviewer’s Comments** | **Response from Author** |
| 1 | Line 123: Provide a reference for “the ARRIVE guidelines”. | References added. Please see lines: 2105 |
| 2 | Fig 1: Pick out one fish image as a representative for each group. | We intend to keep the image in this section as it may give a visual clarification for readers to understand to distinguish the group. |
| 3 | Lines 127-136: Provide more details (such as city and country names) for the companies when necessary. | Revision is done.  Please see lines: 548-613 |
| 4 | Line 150: Provide the full name for TPM and a reference for “RSEM software”. | Revision is done.  Please see lines:627-628 |
| 5 | Lines 159-162: Move this figure to the supplementary section. | The figure is already in the suppelementary section. We delet the figures caption from insdie the text and places after the Reference section. Please see lines: 2577-2580 |
| 6 | Line 164: Rewrite this subtitle. | Revision is done.  Please see lines: 762-763 |
| 7 | Lines 173-174: Change “GB” to “Gb”. What is “CDS predictions”? | Revision is done.  Please see lines: 828-831.  CDS prediction is a fundamental aspect of genomic analysis that involves identifying and annotating the protein-coding regions within a genome, often using computational tools and sequence analysis techniques. |
| 8 | Table 1: Add more data for those missing details. | That's an excellent point regarding missing data. While missing data may be a concern, another crucial consideration is how we handle existing data. We attempted to investigate the best strategies for processing and analyzing the data. We may schedule a more in-depth assessment later, if required. For the time being, we endeavored to keep this effort within its existing scope. |
| 9 | Lines 182-185: Rewrite these sentences. | Revision is done.  Please see lines: 851-853. |
| 10 | Lines 228-230: Why? It doesn’t make sense. | This is a strategic process to narrow down the DEGs for further comparative analysis to find the genes and their pattern of regulation with two exterm groups. |
| 11 | Lines 265-270: The authors may provide more details about these collagen genes, such as transcription difference (ratios) and protein sequences. Is these any premature termination or mismatch of certain important DEG? This may explain the albinism color. | Actually we have compared the CDS of COL1A1α, COL1A1β, COL1A2, COL2A1β, and others collagen genes are also can be compared to *Homo sapiens* with high similarities, but without fish-related CDSs, so it’s not easy to get the CDSs out. Those researches will be studied in the following study and will not be able to be shown in this study. |
| 12 | Discussion: These documents can not support the manuscript even the title, since DEG differences or biomedical uses are missing. | We have imporved some resuts and discussion section fot he manuscript. DEG differences in log2-fold change have been described in the result (lines: 1140-1149). Simialry, related discussion on DEGs difference and biomedical uses have been included in the discussion section (lines: 1195-1274, 1303-1700; 1708-1879). |
| 13 | Supplementary Fig 1: Why are the percentages of complete BUSCOs too low? | In our study, Trinity.oneRow.fa file of this study merges all the transcripts and we had no separating the low and high confidence set. This can be igniting this result. The percentage of complete BUSCOs was found to be lower than expected, most likely due to a number of variables. While the transcriptome assembly was largely robust, it is possible that some low-expressed or tissue-specific orthologs were overlooked. In addition, the sequencing depth was restricted, which may have contributed to insufficient transcriptome coverage. The BUSCO dataset utilized was acceptable for the taxonomic category. [Rivera‐Vicéns, R.E., Garcia‐Escudero, C.A., Conci, N., Eitel, M. and Wörheide, G., 2022. TransPi—a comprehensive TRanscriptome ANalysiS PIpeline for de novo transcriptome assembly. *Molecular Ecology Resources*, *22*(5), pp.2070-2086.) |
|  |  |  |
| **Reviewer 2** | Manuscript can be accepted after minor changes I am of the opinion that if the keywords include words other than the words in the title, it may increase the citation and recognition of the article. Complete line 147 in manuscript file. | Suggested revisitions are completed. Please see lines: 13 and 629-632. |
