# Supplementary material for: Comparative transcriptome analysis of albino northern snakehead (Channa argus) reveals its various collagen-related DEGs in caudal fin cells
Source: PLoS One. 2024 Dec 31;19(12):e0315996. doi: 10.1371/journal.pone.0315996 (PMC11687805; doi:10.1371/journal.pone.0315996)
Supplement: Attachment — Submitted filename: Plose review finaI.docx [file pone.0315996.s006.docx]

Dear Editor

Manuscript can be accepted in current form
