# Supplementary material for: Comparative transcriptome analysis of albino northern snakehead (Channa argus) reveals its various collagen-related DEGs in caudal fin cells
Source: PLoS One. 2024 Dec 31;19(12):e0315996. doi: 10.1371/journal.pone.0315996 (PMC11687805; doi:10.1371/journal.pone.0315996)
Supplement: Attachment — Submitted filename: Response to Reviewers.docx [file pone.0315996.s007.docx]

**Response to the Reviewers:**

Thanks to the reviewers for the comments and suggested revisions to this manuscript. The author's responses are shared below in a table, and the manuscript is revised according to the track changes.

Moreover, we have made an extensive English language improvement in the manuscript as per advice from the Editor.

Apart from that, we did a minor revision to the title of our manuscript. Moreover, we have revised Figure 9 (made scientific names in *italics*) and then uploaded the new image into the system.

Moreover, the lines mentioned (as per with track change mood version) in the table below are according to the revised manuscript.

| **Reviewer 1** | **Reviewer’s Comments** | **Response from Author** |
| --- | --- | --- |
| 1 | Why did the authors select caudal fins? It would be much better to collect muscle samples for transcriptome sequencing and comparative analysis. The authors should provide some descriptions in the Introduction or Discussion. | Thanks for your comment. Our study focuses on the distinctive characteristics of the color morph of C. argus, specifically targeting the unique color differences in their fins. Although previous research has explored various aspects of this species—such as whole genome analysis, genome characterization, molecular diversity, disease and pathogenesis, skin color and albinism, sex chromosomes, feeding behavior, and surimi products—none have investigated the specific color variation in the most manifest part of caudal fins. To address this gap, we collected caudal fin tissues, intentionally excluding scales, skin, or muscle. The golden-finned C. argus stands out from other color morphs, and recent studies have identified fin tissues as a promising source of collagen. We have elucidated this in the Introduction section.  Please see lines: 575 to 836. |
| 2 | Rewrite the subtitles in the Results section, since they look more like those for Materials and Methods | Thanks for your comment. We have revised them all. |
| 3 | Although the authors performed transcriptome sequencing in triplications, they are recommended to validate at least collagen expression by additional qRT-PCR. | Thanks for your comment. We appreciate the reviewer's suggestion to validate collagen expression with additional qRT-PCR. However, due to resource constraints—including limited access to fish samples and funding—we are unable to conduct further experiments. Our study utilized robust transcriptome sequencing in triplicate, ensuring reliable data for collagen expression analysis. Additionally, we have established thorough phylogenetic evidence to support our findings, confirming the evolutionary relevance and consistency of the observed patterns. We are confident that the combination of high-quality transcriptomic data and phylogenetic analysis provides a solid foundation for our conclusions. Moreover, collagen-enriched caudal fins were also been observed in Atlantic salmon by Saveen et al. (2023). Please see lines: 4142 to 4144. |
| 4 | Make sure that related transcriptome data are released. | Thanks for your comment. We have deposited and released the data in NCBI under Bioproject number PRJNA913664 and BioSample number SAMN32303246 with accession number SRX18894708-SRX18894716. Please see lines: 4689 to 4692. |
|  | | |
| **Reviewer 2** | No comments observed | - |

Thank you.

Sincerely yours,

**Shixi Chen, Ph.D. and Fardous Mohammad Safiul Azam, Ph.D.**

College of Life Sciences, Neijiang Normal University, Neijiang, 641100, China.
